# Ankle–foot orthosis with an oil damper versus nonarticulated ankle–foot orthosis in the gait of patients with subacute stroke: a randomized controlled trial

**DOI:** 10.1186/s12984-022-01027-1

**Published:** 2022-05-26

**Authors:** Sumiko Yamamoto, Naoyuki Motojima, Yosuke Kobayashi, Yuji Osada, Souji Tanaka, Aliyeh Daryabor

**Affiliations:** 1 Graduate School, International University of Health & Welfare, 4-1-26 Akasaka, Minato-ku, Tokyo, 107-8402 Japan; 2 Showa University School of Nursing and Rehabilitation Science, 1865 Tohkaichibacho, Midoriku, Yokohama, Kanagawa 226-8555 Japan; 3 Nakaizu Rehabilitation Center, 1523-108 Hiekawa, Izu, Shizuoka 410-2507 Japan; 4 Department of Health and Welfare, Tokushima Bunri University, Nishihamahoji-180, Yamashirocho, Tokushima, 770-8514 Japan; 5 Saiseikai Higashikanagawa Rehabilitation Hospital, 1-13-10 Nishikanagawa, Kanagawa-ku, Yokohama, Kanagawa 221-0822 Japan; 6 School of Rehabilitation, Shahid Beheshti University of Medical Sciences, Velenjak St., Shahid Chamran Highway, Tehran, Iran

**Keywords:** Ankle–foot orthosis, Stroke, Stiffness, Gait, Power absorption

## Abstract

**Background:**

Gait improvement in patients with stroke has been examined in terms of use or non-use of an ankle–foot orthosis (AFO), but the effects of different kinds of AFOs remain unclear. In this study, the effect on gait of using an AFO with an oil damper (AFO-OD), which has plantarflexion stiffness without dorsiflexion resistance, was compared with a nonarticulated AFO, which has both dorsiflexion and plantarflexion stiffness, in a randomized controlled trial.

**Methods:**

Forty-one patients (31 men, 10 women; mean age 58.4 ± 11.3 years) in the subacute phase of stroke were randomly allocated to two groups to undergo gait training for 1 h daily over 2 weeks by physiotherapists while wearing an AFO-OD or a nonarticulated AFO. A motion capture system was utilized to measure shod gait without orthosis at baseline and after training with the allocated AFO. Data analysis focused on the joint kinematics and kinetics, spatial and temporal parameters, ground reaction force, and shank-to-vertical angle. Unpaired *t*-test or Mann–Whitney *U* test was performed to clarify the difference in gait with an AFO between the two AFO groups after training, with a significance level of p = 0.05.

**Results:**

Thirty-six patients completed the study (17 in the AFO-OD group and 19 in the nonarticulated AFO group). The ankle joint was more dorsiflexed in single stance (p = 0.008, effect size r = 0.46) and peak ankle power absorption was larger in stance (p = 0.007, r = 0.55) in the AFO-OD group compared with the nonarticulated AFO group. Peak power absorption varied among patients in the AFO-OD group. Increased dorsiflexion angles were also found at initial contact (p = 0.008, r = 1.51), pre-swing (p = 0.045, r = 0.91), and the swing phase (p = 0.045, r = 0.91) in the AFO-OD group. There was no difference in peak plantarflexion moment, ankle power generation, spatial or temporal parameters, ground reaction force, or shank-to-vertical angle between the two groups.

**Conclusions:**

The results of this study showed that an AFO with plantarflexion stiffness but without dorsiflexion resistance produced greater improvement in ankle joint kinematics and kinetics compared with the nonarticulated AFO, but the results of peak power absorption varied greatly among patients.

*Trial registration* UMIN000028126, Registered 1 August 2017, https://upload.umin.ac.jp/cgi-bin/icdr/ctr_menu_form_reg.cgi?recptno=R000032197

## Introduction

Ankle–foot orthoses (AFOs) are commonly used in clinical practice to improve gait in patients with stroke. Systematic reviews have shown that AFOs can improve gait in these patients by modifying the kinematics of the ankle and knee joints in the stance phase and preventing foot drop in the swing phase [1, 2]. However, the evidence for use of an AFO remains limited because of a lack of sufficient information about the effects of different kinds of AFOs on gait. Considering the effect of different kinds of AFOs on gait, the stiffness of an AFO is considered a key determinant of its functional effect. Here, stiffness is defined as the slope of the curve of moment generated by compression force or resistance versus the ankle joint angle when an AFO is deformed in plantarflexion or dorsiflexion [3, 4]. As AFO stiffness directly affects the ankle joint movement a systematic review showed that the degree of stiffness of an AFO affected ankle kinematics, suggesting that greater stiffness could generally result in a decreased peak ankle joint angle in both plantarflexion and dorsiflexion [5]. Joint kinematics affect the joint kinetics around the ankle joint. Some AFOs have been developed to assist insufficient muscle activity around the ankle joint through AFO stiffness [6, 7]. Therefore, it is important to ascertain the effects of different kinds of AFOs with different stiffness on joint kinematics and kinetics during gait.

There are three main types of passive AFOs available: non-articulated (solid-ankle or rigid), hinged (articulated), and posterior leaf spring (PLS) [8]. Non-articulated and PLS AFOs generate moment by deforming the plastic material of the AFO in both dorsi- and plantarflexion. Most articulated AFOs generate mechanical stiffness by using stoppers, springs, or other devices in plantarflexion but the joints move freely in dorsiflexion. The characteristics of the AFO in plantarflexion affect the gait in the loading response and swing phase, while in dorsiflexion they affect the gait in the mid-to-late stance phase [9]. A previous study compared gait of patients wearing a non-articulated AFO and an articulated AFO, which had a mechanical joint that stopped plantarflexion; therefore, the results included the difference in AFO characteristics both in dorsi- and plantarflexion [10]

An AFO with an oil damper (AFO-OD) was developed based on gait analysis of patients with stroke [11, 12]. The resistance generated by an oil damper slows down the rate of angular change in the ankle joint when it moves to plantarflexion in the loading response. The ankle joint of the AFO-OD moves freely to dorsiflexion. In the present randomized controlled trial, the effects of the non-articulated AFO, which has dorsi- and plantarflexion stiffness, and the AFO-OD, which has plantarflexion stiffness but not in dorsiflexion, on the gait of patients with stroke were compared. This comparison should clarify the difference between AFOs with and without dorsiflexion stiffness. Although there are differences in the characteristics of plantarflexion stiffness between the two AFOs, including the viscosity of the AFO-OD and the elasticity of the nonarticulated AFO, these might be negligible because of the small plantarflexion movement in the loading response.

The dorsiflexion stiffness of an AFO prevents excessive dorsiflexion in the mid-to-late stance phase, which offsets weakness in the plantarflexors [13, 14]; however, large stiffness in dorsiflexion impedes smooth movement of the ankle during the stance phase [1, 9, 15]. The dorsiflexion angle in mid-stance affects the progression of the center of pressure, affecting the ankle joint moment and power [16]. However, to our knowledge, no studies have compared the ankle joint kinetics between AFOs with and without dorsiflexion stiffness and how these differences affect the gait of patients with stroke. Two hypotheses were examined in this study. The first was that the dorsiflexion angle in the mid-to-terminal stance would be smaller when walking with a nonarticulated AFO than with an AFO-OD. The second was that the restricted movement of the ankle joint would induce smaller plantarflexion moment and power around the ankle joint when patients walked with a nonarticulated AFO than with an AFO-OD.

## Methods

### Participants

The participants were patients with subacute stroke (more than 14 and less than 180 days after onset). Patients in the subacute phase were selected for this study because the gait of patients in the chronic phase is affected largely by the use of AFOs in daily life. The participants were inpatients at a rehabilitation center in Japan (Nakaizu Rehabilitation Center) who were walking independently with or without assistive devices and had been prescribed gait training with an AFO. The participants did not use any kind of AFOs in their daily lives. Participants were assessed for eligibility by their rehabilitation physician or physical therapist from April 2018 to October 2019. Exclusion criteria were pre-existing pathology affecting the central nervous system or neuromuscular system and communication problems. In addition, patients who put the nonparetic foot behind the paretic foot (negative step length) during gait without an AFO at baseline were excluded from the analysis because this walking pattern indicates active reduction in paretic propulsion due to severe ankle plantarflexor impairment and no ankle dorsiflexion in paretic stance [17]. All participants underwent gait training under the supervision of physiotherapists. None had previously used an AFO, and all were enrolled in the study when they could walk 10 m under supervision. We calculated the sample size for performing two-way mixed analysis of variance (ANOVA) based on a previous study with peak dorsiflexion angle in the stance phase as the main outcome, assuming a variance of 5.3° and a difference of 5° between the articulated and nonarticulated AFO groups [10]. The significance level was 0.05, and the power was 0.8. Using the G-Power program (Kiel University, Kiel, Germany), the required sample size per group was calculated as 15. To account for data loss, 41 participants were recruited for this study. The study protocol was approved by the ethics committees of the International University of Health & Welfare and Nakaizu Rehabilitation Center. Written informed consent was obtained from all participants prior to their participation in this study.

### Experimental AFO

**Fig. 1:**
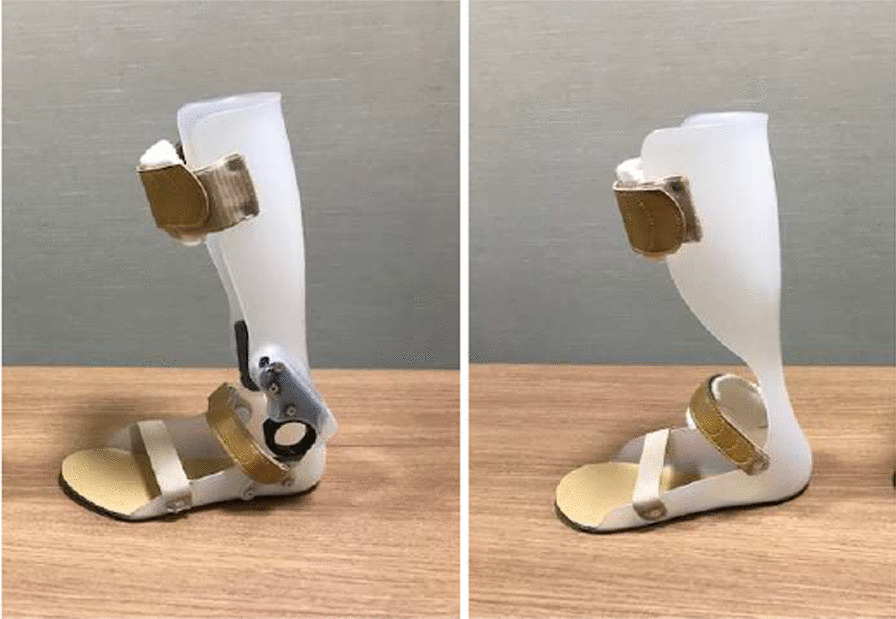
The ankle–foot orthoses used in this study. Left: An ankle–foot orthosis with an oil damper (AFO-OD). Right: A nonarticulated ankle–foot orthosis

The orthoses used in this study were an AFO-OD (GaitSolution; Kawamura Gishi Co. Ltd., Osaka, Japan) and a nonarticulated AFO. The AFO-OD had a mechanical ankle joint with an oil damper (Fig. 1 left) [12] that generates resistance when the ankle joint moves in plantarflexion. The amount of resistance could be changed by rotating a screw at the top of the oil damper from 1 (flexible) to 4 (rigid). In the present study, the screw was set to 3 because this level is used most frequently by patients in the subacute phase of stroke [18]. The AFO-OD allows the ankle joint to move freely in dorsiflexion without any resistance. The nonarticulated AFO used in this study had no mechanical joint and an ankle trim line behind the malleoli (Fig. 1 right). The initial ankle joint angle was set to neutral, meaning that the shank was vertical to the floor in both AFOs. Neither type of AFO were custom-made, so we prepared 6 for each group, namely, right and left AFOs in each of the three available sizes (small, medium, and large).

The stiffness of all AFOs was measured using a goniometer, a digital force gauge (Imada Co. Ltd., Toyohashi, Japan), and a metronome. Because of differences in the inherent characteristics of plastic, which has elasticity, and the oil damper, which has viscosity, the measurement was static for the nonarticulated AFO and dynamic for the AFO-OD. The sole of the AFO was fixed on a table and the shank was pulled perpendicularly by a wire attached to the shank. The wire was pulled to 5° in both directions when obtaining measurements for the nonarticulated AFO. When recording measurements for the AFO-OD, the wire was pulled once per second and 4 times per second in plantarflexion and the peak force was recorded by the force meter. Each speed corresponds to 5°/s and 20°/s [12]. Table 1 shows the characteristics of the AFOs as the average of the right and left AFOs in each size. As shown in Table 1 dorsiflexion stiffness of the nonarticulated AFOs ranged from 1.75 to 1.92 Nm/°. In a systematic review that investigated the impact of AFO stiffness on gait, the reported range of stiffness in the included studies was 0.02–8.17 Nm/° [5]. Another study comparing four kinds of AFOs reported a stiffness range of 0.2 to 2.0 Nm/° [19]. Based on these studies, the dorsiflexion stiffness of the nonarticulated AFO used in this study may be considered moderate to relatively stiff.

**Table 1:** Characteristics of the AFOs used in this study

	AFO-OD			Nonarticulated AFO		
	Small	Medium	Large	Small	Medium	Large
Weight (g)	395	430	500	310	360	385
Calf height (cm)	32.0	35.0	37.0	32.0	35.0	37.0
Foot length (cm)	22.5	24.5	27.0	22.5	24.5	27.0
Width (cm)	N.A	N.A	N.A	6.7	6.7	7.0
Wall thickness (cm)	0.3	0.3	0.3	0.3	0.3	0.3
Plantarflexion stiffness	3.12 Nm*	3.38 Nm*	3.08 Nm*	3.19 Nm/°	3.16 Nm/°	3.12 Nm/°
	5.46 Nm**	5.81 Nm**	5.74 Nm**			
Dorsiflexion stiffness	N.A	N.A	N.A	1.80 Nm/°	1.75 Nm/°	1.92 Nm/°

AFO-OD, ankle–foot orthosis with an oil damper; nonarticulated AFO, nonarticulated ankle–foot orthosis; N.A., not available

^*^Peak resistive moment at slow speed

**Peak resistive moment at fast speed

### Study protocol and randomization

This single-center, non-blinded, randomized controlled, parallel group trial was registered in the University hospital Medical Information Network registry (UMIN000028216). The participants were randomly allocated to the AFO-OD group or the nonarticulated AFO group by a physiotherapist in order of participation with an allocation ratio of 1:1. First, shod gait with no AFO was measured at each participant’s self-selected walking speed along an 8-m walkway. Gait was measured using a motion capture system with 8 Vicon MX cameras (VICON Motion System, Ltd., Oxford, UK) and 6 AMTI force plates (Advanced Mechanical Technology, Inc., Phoenix, AZ). Reflective markers were attached to the participants’ bodies in accordance with the Plug-In Gait model. Additional markers were used on the medial side of the knee and ankle joints to precisely calculate the joint centers. Marker trajectories and force plate data were captured at a sampling frequency of 100 Hz. The participants wore shoes (V-step; Pacific Supply, Osaka, Japan) that were of a suitable size for each condition with and without an AFO. The use of a cane was permitted, but it had to be used in a consistent manner when walking without an AFO and with either type of AFO.

Then, the participants started gait training sessions using the allocated AFOs for 1 h daily over 2 weeks under the supervision of a team of physiotherapists. The participants used each kind of AFO just during gait training. They also participated in general physiotherapy, including range of motion exercises, balance training, and muscle training. There were no differences in the physiotherapy processes between the two AFO groups. After 2 weeks of training, gait with the allocated AFOs was measured using the same procedure.

### Data analysis and outcome measures

Marker trajectories and force plate data were low-pass filtered by a second-order Butterworth filter with cutoff frequencies of 6 and 18 Hz, respectively. The gait cycle time and duration of each gait phase were defined using force plate data. The trajectories of heel markers were used if the participant walked with a cane on their nonparetic side. Given that participants were in the subacute phase of stroke, they did not show a heel rise in the paretic single stance. Therefore, the gait cycle was defined as the loading response, single stance, pre-swing, and swing phases of the paretic limb. Joint kinematics and kinetics were calculated using an inverse dynamic model. The primary outcome measure was peak dorsiflexion ankle joint angle in single stance, and the secondary outcomes were peak ankle plantarflexion moment and peak ankle power in the stance phase. The following parameters were calculated as other outcome measures: ankle joint angles in other gait phases; knee and hip joint kinematics and kinetics; spatial and temporal parameters, including velocity, step length, and duration of each gait cycle; and the anterior and posterior components of the ground reaction force. The angle of inclination of the shank segment relative to the vertical (shank-to-vertical angle) in the sagittal plane was calculated in view of its importance when evaluating gait with an AFO [20]. Step length was normalized by each participant’s body height, and ground reaction force, joint moment, and power were normalized by the participant’s body weight. Ground reaction forces as well as kinematic and kinetic data were obtained for the paretic limb. All calculations were performed using Visual 3D software version 6 (C-Motion Inc., Kingston, ON, Canada).

### Statistical analysis

All gait parameters are presented as the average of at least three gait cycles for each condition. The data were assessed for normality by the Shapiro–Wilk test. For the baseline data, differences between the two AFO groups in all gait parameters without AFOs were compared using the unpaired *t*-test or the Mann–Whitney *U* test. Gait with each type of AFO after 2 weeks of gait training was compared using the unpaired *t*-test or Mann–Whitney *U* test. The correlation coefficient was also calculated between parameters that showed significant difference between groups. A p-value of < 0.05 was considered statistically significant. All statistical analyses were performed using SPSS for Windows version 25 (IBM Corp., Armonk, NY).

## Results

**Fig. 2:**
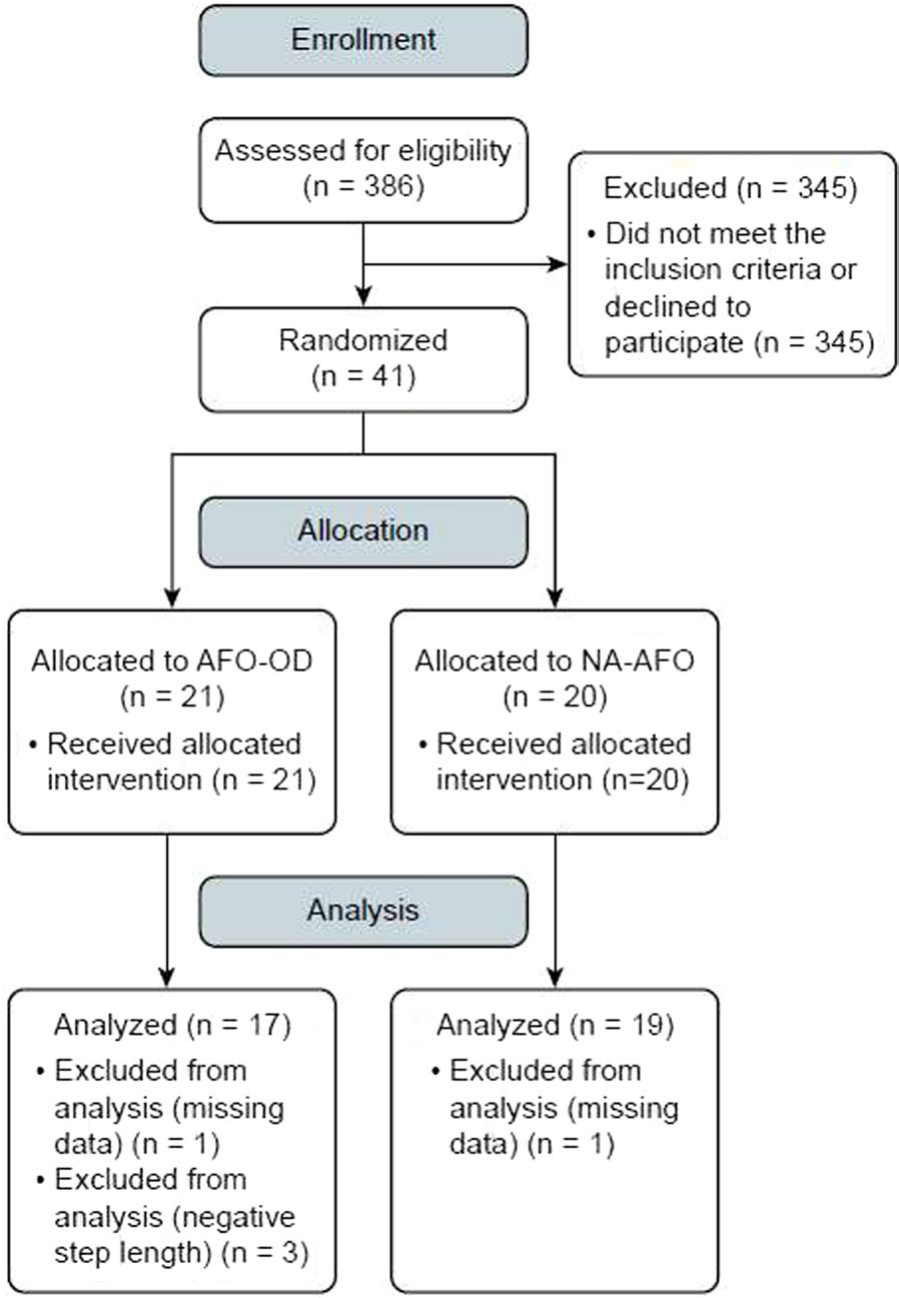
Consolidated Standard of Reporting Trials (CONSORT) flowchart

The study flowchart is shown in Fig. 2. Recruitment started in April 2018 and was stopped in October 2019 because the required sample number was obtained. One participant in each group was withdrawn because of missing data during the study period and three participants in the AFO-OD group were excluded from the analysis because of placement of the nonparetic foot behind the paretic foot at baseline. This left data for 36 participants for analysis (AFO-OD group, n = 17; nonarticulated AFO group, n = 19). There was no significant difference between the groups in terms of age, body height, and weight or days since stroke onset at baseline (Table 2). Eleven of the 17 participants in the AFO-OD group and 15 of the 19 in the nonarticulated AFO group used a cane. No unintended effects were found during the participation.

**Table 2:** Patient characteristics

	AFO-OD (n = 17)	Nonarticulated AFO (n = 19)	
Sex	Male: 13, Female: 4	Male: 15, Female: 4	
Age, years	56.2 (12.9)	60.5 (9.7)	ns
Body height, cm	167.2 (7.6)	165.5 (7.6)	ns
Body weight, kg	58.7 (9.5)	64.5 (11.6)	ns
Diagnosis	Cerebral hemorrhage: 10, Cerebral infarction: 7	Cerebral hemorrhage: 7, Cerebral infarction: 12	
Paretic side	Right: 8, Left: 9	Right: 11, Left: 8	
Days since onset	69.7 (41.6), Min: 28, Max: 145	65.8 (39.5), Min: 22, Max: 147	ns
Brunnstrom stage in lower extremities	II: 2, III: 7, IV: 3, V: 5	II: 0, III: 11, IV: 5, V: 3	
Manual ROM test of ankle joint	0°: 1, 5°: 8, 10°: 5, 15°: 3	− 5°: 1, 0°: 2, 5°: 4, 10°: 6, 15°: 5, 20°: 0, 25°: 1	
Modified Ashworth Scale	0: 5, 1: 3, 1+: 4, 2: 5	0: 7, 1: 4, 1+: 6, 2: 2	
Use of cane	Yes: 11, No: 6	Yes: 15, No: 4	

Mean (standard deviation)

AFO-OD, ankle–foot orthosis with an oil damper; nonarticulated AFO, nonarticulated ankle–foot orthosis; ns, not significant; ROM, range of motion

In the baseline comparison, no difference was found in any gait parameter between the two AFO groups (p > 0.05). The results for the comparison of joint kinematics and kinetics between the two types of AFO worn after training are shown in Table 3. For the primary outcome measure, the ankle joint was more dorsiflexed in single stance (mean difference 5.35°; 95% CI 1.50–9.19, p = 0.008, effect size r = 0.46) in the AFO-OD group than in the nonarticulated AFO group. As for the secondary outcomes, the peak ankle power absorption in stance was larger in the AFO-OD group than in the nonarticulated AFO group (mean difference 0.304 W/kg; 95% CI 0.092–0.516, p = 0.007, r = 0.55). However, there was no difference between the two AFO groups in peak ankle plantarflexion moment and peak ankle power generation in the stance phase.

**Table 3:** Comparison of joint kinematics and kinetics between the two AFO groups after training

	AFO-OD	Nonarticulated AFO	p-value	Effect size r
Ankle angle				
Initial contact (°)^#^	1.98 (5.75)	− 2.48 (4.32)	0.008**	1.51
Peak plantarflexion in loading response (°)^#^	− 1.25 (6.15)	− 6.54 (4.26)	0.182	
Peak dorsiflexion in single stance (°)	6.73 (6.54)	1.39 (4.76)	0.008**	0.46
Peak plantarflexion in pre-swing (°)^#^	3.06 (7.19)	− 1.07 (3.78)	0.045*	0.91
Peak dorsiflexion in swing (°)^#^	5.46 (7.28)	0.05 (4.17)	0.045*	0.91
Ankle angular velocity				
Peak plantarflexion in loading response (°/s)^#^	− 36.41 (23.53)	− 34.20 (21.06)	0.869	
Peak dorsiflexion in single stance (°/s)^#^	32.54 (28.81)	24.57 (11.54)	0.129	
Peak plantarflexion in pre-swing (°/s)^#^	− 31.36 (19.81)	− 33.36 (29.53)	0.869	
Ankle moment				
Peak dorsiflexion in loading response (Nm/kg)^#^	− 0.07 (0.08)	− 0.08 (0.07)	0.999	
Peak plantarflexion in stance (Nm/kg)	0.92 (0.30)	0.74 (0.21)	0.054	
Ankle power				
Peak absorption in stance (W/kg)	− 0.58 (0.37)	− 0.28 (0.17)	0.007**	0.55
Peak generation in stance (W/kg)	0.32 (0.22)	0.20 (0.20)	0.111	
Knee angle				
Initial contact (°)^#^	14.31 (4.03)	9.91 (9.46)	0.045*	0.91
Peak flexion in loading response (°)	21.28 (6.25)	18.63 (7.42)	0.054	
Peak extension in single stance (°)^#^	7.39 (6.77)	5.81 (9.68)	0.504	
Knee moment				
Peak extension in loading response (Nm/kg)	0.44 (0.25)	0.34 (0.15)	0.136	
Peak extension in single stance (Nm/kg)	0.26 (0.33)	0.23 (0.20)	0.796	
Peak flexion in single stance (Nm/km)	− 0.29 (0.31)	− 0.26 (0.21)	0.735	
Hip angle				
Initial contact (°)	23.36 (7.40)	20.08 (5.42)	0.132	
Peak extension in single stance (°)	6.01 (8.12)	4.48 (8.77)	0.593	
Hip moment				
Peak extension in loading response (Nm/kg)	0.60 (0.28)	0.67 (0.30)	0.479	
Peak flexion in single stance (Nm/kg)	− 0.59 (0.64)	− 0.79 (0.52)	0.309	
Peak flexion in pre-swing (Nm/kg)	− 0.88 (0.96)	− 0.80 (0.24)	0.752	

Angle, dorsiflexion, flexion+; Internal moment, plantarflexion, extension +

Mean (standard deviation) for normally distributed data; median (interquartile range) for non-normally distributed data; ns, not significant

AFO-OD, ankle–foot orthosis with an oil damper; nonarticulated AFO, nonarticulated ankle–foot orthosis

^*^p < 0.05; **p < 0.01; ^#^Not normally distributed

As for the other outcome measures, compared with the nonarticulated AFO group, the ankle joint in the AFO-OD group was more dorsiflexed at initial contact (p = 0.008, r = 1.51), in pre-swing (p = 0.045, r = 0.91), and in the swing phase (p = 0.045, r = 0.91). The knee joint was more flexed at initial contact in the AFO-OD group than in the nonarticulated AFO group (p = 0.045, r = 0.91). The mean difference and 95% CI are not shown because these parameters were not normally distributed. There was no difference between the two AFO groups in spatial or temporal parameters, ground reaction forces, or shank-to-vertical angle (Table 4).

**Table 4:** Comparison of temporal and spatial factors, ground reaction forces, and shank-to-vertical angle between the two AFO groups at post-training

	AFO-OD	Nonarticulated AFO	p-value
Spatial and temporal			
Velocity (m/s)^#^	0.332 (0.189)	0.367 (0.120)	0.504
Step length (paretic to non-paretic)/height	0.200 (0.083)	0.189 (0.059)	0.657
Step length (non-paretic to paretic)/height	0.209 (0.073)	0.202 (0.061)	0.630
Cycle time (s)	1.886 (0.623)	1.854 (0.563)	0.622
Loading response time (s)^#^	0.308 (0.242)	0.317 (0.129)	0.568
Single stance time (s)	0.428 (0.104)	0.429 (0.118)	0.982
Pre-swing time (s)^#^	0.425 (0.525)	0.386 (0.360)	0.987
Swing time (s)^#^	0.593 (0.152)	0.583 (0.117)	0.279
Ground reaction force			
Peak posterior (N/kg)	− 0.904 (0.462)	− 0.857 (0.207)	0.702
Peak anterior (N/kg)	0.449 (0.246)	0.495 (0.305)	0.622
Shank-to-vertical angle			
Initial contact (°)	− 2.793 (5.089)	− 5.772 (5.397)	0.099
Initial contact of non-paretic limb (°)	14.209 (6.330)	10.376 (4.992)	0.291
Range in single stance (°)	8.088 (4.623)	5.642 (4.888)	0.133

Forward inclination of shank-to-vertical angle+

Mean (standard deviation) for normally distributed data; median (interquartile range) for non-normally distributed data; ns, not significant

AFO-OD, ankle–foot orthosis with an oil damper; nonarticulated AFO, nonarticulated ankle–foot orthosis

^*^p < 0.05; **p < 0.01; ^#^Not normally distributed

**Fig. 3:**
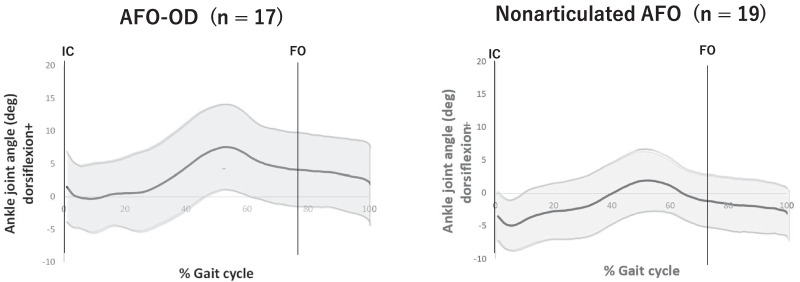
Graph showing the average angle of the ankle joint during one gait cycle in each AFO group after training (AFO-OD group, n = 17; nonarticulated AFO group, n = 19). Bold lines indicate average values and non-bold lines denote standard deviations. AFO, ankle–foot orthosis; AFO-OD, ankle–foot orthosis with an oil damper; IC, initial contact; FO, foot off

**Fig. 4:**
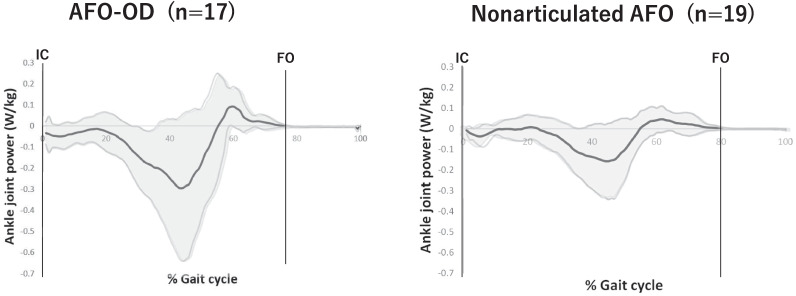
Graph showing average ankle power during one gait cycle in each AFO group after training (AFO-OD group, n = 17; nonarticulated AFO group, n = 19). Bold lines denote average values and non-bold lines denote standard deviations. AFO, ankle–foot orthosis; AFO-OD, ankle–foot orthosis with an oil damper; IC, initial contact; FO, foot off

Figure 3 shows the mean ankle joint angle and Fig. 4 shows the ankle power in one gait cycle for each AFO group. As these figures show, the ankle joint was more dorsiflexed throughout the gait cycle and dorsiflexion in the stance phase was larger in the AFO-OD group than in the nonarticulated AFO group. Figure 4 shows that the power absorption was high with a large standard deviation in the AFO-OD group and was low with a small standard deviation in the nonarticulated AFO group.

**Fig. 5:**
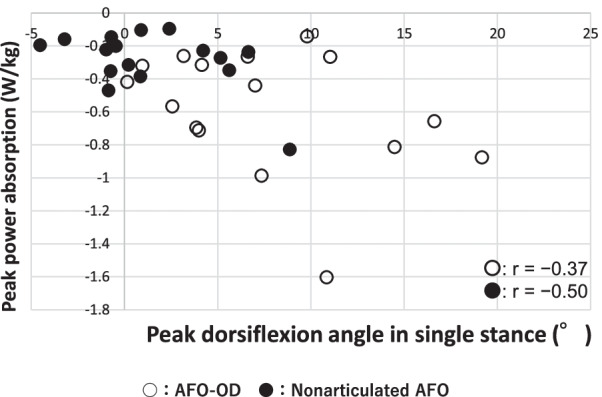
Relationship between peak angle of ankle dorsiflexion and peak power absorption at the ankle during gait in the two AFO groups. AFO, ankle–foot orthosis; AFO-OD, ankle–foot orthosis with oil damper

Figure 5 shows the relationship between the peak dorsiflexion angle in single stance and peak ankle power absorption in stance for each type of AFO. Each mark represents an individual participant (white, AFO-OD group; black, nonarticulated AFO group). Peak dorsiflexion angle and peak power absorption were large and distributed across a wide range in the AFO-OD group; there was no correlation between these parameters in the AFO-OD group (r = − 0.37, p = 0.15). Although small, these parameters were correlated with each other (r = − 0.50, p = 0.043) in the nonarticulated AFO group.

## Discussion

We hypothesized that the peak ankle joint angle in dorsiflexion in single stance as well as the ankle plantarflexion moment and power would be smaller in the nonarticulated group. Our results partially supported these hypotheses: the peak ankle dorsiflexion angle in single stance was significantly smaller in the nonarticulated AFO group than in the AFO-OD group (1.39° vs 6.73°, p = 0.008), and the peak absorption of ankle power was also significantly smaller in the nonarticulated AFO group than in the AFO-OD group (− 0.28 W/kg vs − 0.58 W/kg, p = 0.007). However, there were no differences between the two AFO groups in ankle plantarflexion moment and peak power generation.

In terms of kinematics, the ankle joint during gait was more dorsiflexed at initial contact and in the single stance, pre-swing, and swing phases with the AFO-OD than with the nonarticulated AFO, which is consistent with previous findings [10, 21]. Systematic reviews have indicated that many previous studies focused on the peak dorsiflexion angle needed in the swing phase to keep clearance [1, 2]. The median of the peak dorsiflexion angle in the swing phase was 5.46° in the AFO-OD group and 0.05° in the nonarticulated AFO group. One of the important findings of the present study was that the peak dorsiflexion angle in the swing phase was larger with the AFO-OD than with the nonarticulated AFO.

The difference in moment generated by the two types of AFOs in plantarflexion was thought to occur in the loading response during gait. There was no difference in peak plantarflexion angle or angular velocity during the loading response between the two AFO groups. In terms of the moment in plantarflexion, the average peak plantarflexion moment generated by the nonarticulated AFO was approximately 12 Nm, with 4° plantarflexion, from − 2.48° to − 6.54°, as shown in Table 3, in loading response and 3.16 Nm/° for the AFO characteristics. We did not obtain the moment for the AFO-OD at 36°/s in plantarflexion but estimated it to be less than 10 Nm based on the results shown in Table 1. The moment to plantarflexion during loading response was similar in the two AFO groups.

The degree of stiffness in dorsiflexion provided by different types of AFO can affect the kinetics of the ankle joint in the mid-to-late stance phase [1, 9, 15]; however, to our knowledge, this had not been confirmed in any previous studies. Table 3 and Fig. 4 show that the power absorption was larger when walking with the AFO-OD than when walking with the nonarticulated AFO. Power absorption is related to eccentric contraction of the plantarflexors in mid-stance [16] and is obtained by multiplying the plantarflexion moment by the angular velocity of ankle dorsiflexion [22]. In this study, we found no significant difference in peak angular velocity of ankle dorsiflexion between the groups. Although the difference in peak plantarflexion moment in the stance phase was not significant (p = 0.054), but the main contributor to the difference in power absorption appears to be the larger plantarflexion moment in the AFO-OD group.

In the nonarticulated AFO group, the power absorption and plantarflexion moment values included both biological power and moment and AFO-generated power and moment [11, 23]. The average value of the moment for the nonarticulated AFO was approximately 2.5 Nm, calculated as 1.39° (peak dorsiflexion angle in single stance, as shown in Table 3) multiplied by 1.82 Nm/° (mean dorsiflexion stiffness of three nonarticulated AFOs, as shown in Table 1). However, the peak plantarflexion moment in stance was 47.7 Nm, calculated as 0.74 Nm/kg multiplied by average body weight 64.5 kg. The moment generated by the elasticity of the nonarticulated AFO was not large in the stance phase. This finding is in line with that in previous studies in which the AFO-generated moment during gait was estimated using a nonarticulated AFO and a testing machine [23]. In previous studies, the moment provided by an AFO was thought to be important to compensate for insufficient activity of the plantarflexors in mid-to late stance [13, 14, 24]. However, the results of the present study show that moment provided by a moderate to relatively stiff nonarticulated AFO is not large compared with the biological moment. The moment and power in the AFO-OD did not include the effect of the AFO directly because the AFO-OD did not provide any resistance in dorsiflexion. Further study is needed to determine the activity of the plantarflexors with different types of AFO.

As shown in Fig. 5, the peak power absorption was distributed in a narrow range and related to the peak angle of dorsiflexion in the nonarticulated AFO group because of the assisted dorsiflexion provided by the AFO. In the AFO-OD group, the peak power absorption was large but variable because dorsiflexion was not assisted by the AFO. The variation in ankle power absorption among patients in the AFO-OD group suggests a marked difference in plantarflexor activity between patients. The activity of the plantarflexors in stance is important in terms of stability and propulsion in normal gait [22, 25] as well as in the gait of patients with stroke [6, 7]. Therefore, an AFO-OD, which does not provide dorsiflexion resistance, would be beneficial in some patients but not others. As mentioned in previous studies [9, 26], the stiffness of the AFO should be adjusted in dorsiflexion and plantarflexion separately; however, this would be difficult for a nonarticulated AFO, where both dorsiflexion and plantarflexion are affected by the shape and thickness of the plastic materials used [27, 28].

Increased power absorption in the stance phase might affect power generation in late stance. The plantarflexors store energy and generate propulsive force in late stance [22, 25]. In this study, there was no difference in the ankle power generation between the two AFO groups. In normal gait, the ankle joint shows maximum plantarflexion in pre-swing [16] but the amount of plantarflexion was small in the AFO-OD group (Table 3). Other studies have also found a decrease in the power generated at the ankle when the AFO resisted plantarflexion [9, 29]. However, the ability to generate power can be improved by gait training using an AFO [30]. The long-term effect of gait training with an AFO on muscle activity requires further study.

This study has several limitations. The degree of stiffness of the AFO-OD in plantarflexion and of the nonarticulated AFO in plantar and dorsiflexion was the same for each participant. The fit of the AFOs, which were not custom-made, and weight differences between the two kinds of AFOs might have affected the results. The use of custom-made AFOs with optimized resistance might have had a positive effect on both AFO groups. There was a difference in the stiffness to plantarflexion between the two kinds of AFOs used in this study in terms of viscosity and elasticity. Most participants in the two groups used a cane on their nonparetic side. The use of a cane might have had an effect on the velocity and joint kinematics [31], but these participants could not walk without a cane. Finally, this study was conducted at a rehabilitation center, and thus further study in different settings will be necessary to ascertain the generalizability of the results.

## Conclusion

This randomized controlled trial compared the effect of a nonarticulated AFO, which had stiffness both in dorsi- and plantarflexion, with an AFO-OD, which had stiffness in plantarflexion but not in dorsiflexion. The results of this study showed that the AFO-OD improved ankle joint kinematics and kinetics more than a nonarticulated AFO, but the results showed large variation among patients.

## Data Availability

The raw data were generated at Nakaizu Rehabilitation Hospital. Derived data supporting the findings of this study are available from the corresponding author S.Y. on request.

## Abbreviations

AFO: Ankle–foot orthosis
AFO-OD: Ankle–foot orthosis with an oil damper
PLS: Posterior leaf spring
